# Diagnostic investigation, intervention, and outcome for post-subtotal gastrectomy patients who present with jaundice

**DOI:** 10.3389/fmed.2025.1485442

**Published:** 2025-02-12

**Authors:** Duan Wang, Liang Zhu, Fanyi Kong, Yingyu Pan, Wei Liu, Xuan Wang, Weidong Pan, Jian Cao, Qiang Xu, Dong Wu

**Affiliations:** ^1^Department of Gastroenterology, Peking Union Medical College Hospital, Beijing, China; ^2^Department of Gastroenterology, Tibet Autonomous Region People's Hospital, Lhasa, China; ^3^Department of Radiology, Peking Union Medical College Hospital, Beijing, China; ^4^Department of General Surgery, Peking Union Medical College Hospital, Beijing, China; ^5^Clinical Epidemiology Unit, Peking Union Medical College Hospital, Chinese Academy of Medical Sciences and Peking Union Medical College, Beijing, China

**Keywords:** obstructive jaundice, subtotal gastrectomy, imaging, intervention, outcome

## Abstract

**Purpose:**

Endoscopic retrograde cholangiopancreatography (ERCP) is a useful diagnostic and interventional tool in patients with obstructive jaundice. In patients who had subtotal gastrectomy, however, the implementation of ERCP has become more difficult. This study aims to investigate the accuracy of contrast-enhanced CT, MRI/MRCP and PET/CT in lesion localization, characterization, and extent evaluation in post-subtotal gastrectomy patients who present with obstructive jaundice. The interventional methods for biliary drainage, their success rate and patient outcome were also investigated.

**Methods:**

Electronic medical records were reviewed to identify patients hospitalized for obstructive jaundice at Peking Union Medical College Hospital, who had previously undergone subtotal gastrectomy. The clinical information, imaging and interventional examination data of those patients were retrospectively collected.

**Results:**

Between 2018 and 2023, 36 patients with previous subtotal gastrectomy were hospitalized for ob-structive jaundice at our hospital. The majority of lesions were malignant, including 19 gastric cancer recurrence (47.5%), and 12 other malignancies (30.0%). Benign lesions included inflammatory biliary stricture, biliary stones, and IgG4-related disease. The three imaging modalities had similar performance in lesion localization and characterization, whereas PET/CT showed higher accuracy compared to MR and CT in detecting extensive disease (92.8% vs. 83.3% vs. 60.0%). Percutaneous transhepatic cholangial drainage was applied more frequently than ERCP and surgery (69.4% vs. 25.0% vs. 5.5%), and there was no significant difference concerning technical and clini-cal success rate and complication.

**Conclusion:**

Gastric cancer recurrence and newly-developed pancreaticobiliary malignancies were the main causes of obstructive jaundice in patients who had subtotal gastrectomy. PET/CT was superior to MRI/MRCP and contrast-enhanced CT in determining lesion extensiveness. Percutaneous transhepatic cholangial drainage (PTCD) was the preferred method for managing obstructive jaundice. Despite the effectiveness of interventions, a significant number of patients experienced short-term disease progression.

## 1 Introduction

Obstructive jaundice is a condition resulting from biliary stricture and partial or complete mechanical obstruction of the intrahepatic or extrahepatic bile ducts, leading to pathophysiological dysfunction in multiple organ systems (1). Both benign and malignant disease entities could cause obstructive jaundice. Common benign entities included surgical trauma, gallstone disease, duodenal ulcer and chronic pancreatitis (2), and common malignant entities included cholangiocarcinoma, gallbladder cancer, pancreatic cancer, primary/metastatic liver cancer, and metastatic lymph nodes (3). It is clinically important to identify the exact cause and location of obstructive jaundice, which affects the targeted treatment planning, and prognosis evaluation.

Endoscopic retrograde cholangiopancreatography (ERCP) is an important diagnostic and interventional tool for patients with obstructive jaundice, providing a one-stop diagnosis and treatment approach. Besides evaluating lesion location and extent and se-verity of biliary stricture with the cholangiogram, cytologic brushing, and pathological sampling could also be performed. Stone removal and biliary stent placement could also be done during the procedure. However, in patients with a history of subtotal gastrectomy, the position of the duodenal papilla and the direction of sphincterotomy deviate from the normal anatomy, especially following Billroth II (B-II) and Roux-en-Y anastomosis. Consequently, ERCP procedures become more challenging and hazardous (4), necessitating greater reliance on alternative imaging modalities and interventional procedures for diagnosis and management in such patients (5).

Percutaneous transhepatic cholangial drainage (PTCD) and ERCP play crucial roles in the palliative treatment of malignant obstructive jaundice, biliary decompression, and preoperative drainage, with their efficacy and safety well-established. Studies have demonstrated that PTCD has a higher success rate compared to ERCP, along with better improvements in jaundice resolution and liver function (6). However, PTCD may also lead to a higher incidence of complications, primarily associated with catheter displacement, and improper care. Prolonged catheter placement could further impact the patient's quality of life.

To our knowledge, there were few reports on the clinical and radiologic characteristics of patients with obstructive jaundice who have a history of subtotal gastrectomy, and the existing literature mainly consisted of case reports on novel endoscopic techniques or surgical skills. Little is known about the disease spectrum, the diagnostic lesions causing obstructive jaundice with different imaging modalities, and the efficacy of different interventional measures in this special patient cohort. This study aims to gain a comprehensive understanding of the spectrum of etiology, advantages and limitations of commonly used diagnostic modalities, and prognosis of such patient, which may aid in clinical decisions.

## 2 Materials and methods

### 2.1 Study cohort

**Figure 1:**
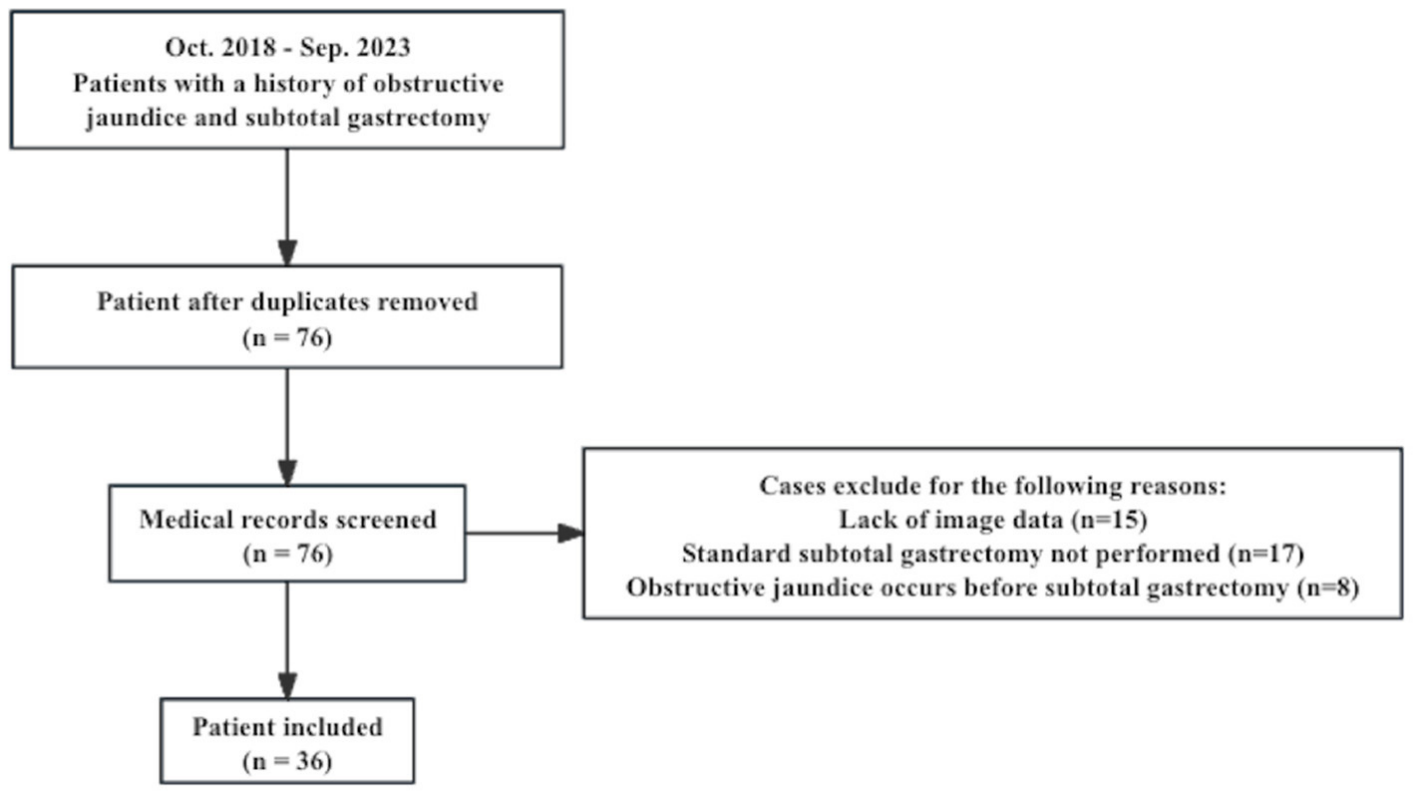
Flowchart of the patient selection.

This study received approval from the Ethics Committee of Peking Union Medical College Hospital (PUMCH), and patient informed consent was waived. This study retrospectively collected clinical, imaging, and interventional examination data from patients hospitalized for obstructive jaundice at a single tertiary medical center, who had previously undergone subtotal gastrectomy. The inclusion criteria were: (1) patient was hospitalized at Peking Union Medical College Hospital between 2018 and 2023 for obstructive jaundice; (2) patient had a history of subtotal gastrectomy surgery; (3) patient underwent at least one of the following pre-treatment imaging examinations for obstructive jaundice: contrast-enhanced CT, MRI/MRCP, PET/CT. The exclusion criteria were: (1) incomplete clinical data; (2) patient didn't fulfill the diagnostic criteria of obstructive jaundice; (3) lack of a clear etiological diagnosis for obstructive jaundice at discharge. The flowchart of patient selection is shown in Figure 1.

### 2.2 Clinical and imaging data collection

Baseline data were collected, including gender, age, time and type of major gastric resection surgery, onset time of obstructive jaundice, and clinical symptoms. Laboratory test results were collected, including tumor markers and bilirubin levels.

Contrast-enhanced CT, MRI/MRCP, and PET/CT imaging data and reports were retrieved from the picture archiving and communication system (PACS) of PUMCH.

The initial diagnosis of patients' obstructive jaundice was determined through a multidisciplinary approach involving experienced radiologists, gastroenterologists, and pathologists, based on all the available clinical, imaging and pathology results from cytological brushing, biopsy, or surgical specimens during patient hospitalization. In this retrospective study, two additional senior gastroenterologists were invited to check all the medical records and taking into account the patients' clinical course in case incorrect diagnosis was made according to all the data available. The major imaging findings were recorded by two senior radiologists in a structured manner, including mass, biliary strictures, wall thickening of the bile duct, lymphadenopathy, ascites, peritoneal im-plants, bile duct stones, and pancreatic diffuse enlargement. They made radiological diagnoses based on the above imaging findings for the location of the lesion causing obstructive jaundice (within the intra-pancreatic segment of the common bile duct or above), the type of the lesion (benign or malignant), and the extent of the disease (local disease or with distant metastasis). The disease extent was defined as the local staging within the CT/MRI abdominal scan range, which evaluates the involvement of adjacent organs, blood vessels, peritoneum, and lymph node metastasis. If the opinions of two radiologists differ, they will discuss the discrepancy until a consensus is reached.

The interventional method and operation details were collected from patient medical records. The technical success of ERCP was defined as the achievement of successful duodenal stump intubation and stone removal. The technical success of PTCD was defined as an unobstructed drainage tube with a drainage volume of >5 ml (7). Clinical success of biliary drainage was defined as at least a 30% reduction of total serum bilirubin from baseline after technical success (8). Recurrence of Gastric Cancer: In patients who had undergone subtotal gastrectomy for gastric cancer, obstructive jaundice can occur due to the compression of the intrahepatic and extrahepatic bile ducts by metastatic gastric cancer. Occurrence of other types of malignancies: Among patients who had previously undergone subtotal gastrectomy for benign diseases or gastric cancer, obstructive jaundice may be caused by a stenosis of the bile duct due to other types of malignancies.

Patient status at discharge and time to disease progression were recorded based on hospital records and outpatient follow-up data. Disease-free survival rate was defined as the fraction of patients observed for a duration exceeding 3 months within an out-patient clinic context, who have remained free from recurrent obstructive jaundice or tumor advancement by the follow-up period's termination. Disease-free survival time was defined as the time from improvement of obstructive jaundice to recurrence of obstructive jaundice/progression of tumor.

### 2.3 Statistical analysis

Data analysis was conducted using SPSS software version 27.0. Results were presented as mean, median, range, and proportion. Descriptive data was displayed in terms of percentages and means with standard deviations when they were normally distributed. For measurement data not conforming to normal distribution, values are expressed as median with range. The sensitivity, specificity, and accuracy of contrast-enhanced CT, MRI/MRCP and PET/CT were calculated with the 2 × 2 contingency table and compared with Chi-square test and Fisher's exact test. The efficacy and Complication Rates of ERCP and PTCD were compared with Chi-square test. A double-sided *P* < 0.05 was considered significant difference.

## 3 Results

### 3.1 Patient characteristics

A total of 36 patients who had previously undergone subtotal gastrectomy were admitted at Peking Union Medical College Hospital for obstructive jaundice between October 2018 and September 2023. The patient characteristics are summarized in Table 1. The majority of the patients were male (30/36, 83.3%), with an average age of 63.3 ±11.0 years. Upon admission, the median total bilirubin levels were 127.1 μmol/L (range, 37.4–488.9), and the median direct bilirubin levels were 94.3 μmol/L (range, 20.1–426.4). Elevated serum tumor markers were observed in 23 patients (79.3%), including elevated CEA in 16 patients (16/31, 51.6%), elevated CA19-9 in 21 patients (21/28, 75.0%), elevated CA242 in 8 patients (8/20, 40.0%) and elevated CA125 in 7 patients (7/17, 41.2%). The median time between the gastrectomy and the development of obstructive jaundice was 3 years (range, 0.2–53.0 years). The most common surgical procedure the patients received was Billroth II subtotal gastrectomy (*n* = 15, 47.1%), followed by Roux-en-Y (*n* = 9, 25.0%), and Billroth I subtotal gastrectomy (*n* = 6, 16.7%). In 6 patients (16.7%) the types of gastrectomy were unspecified. Twenty-six patients (72.2%) underwent subtotal gastrectomy for gastric cancer, and ten patients (27.8%) underwent the surgery for gastric ulcer/perforation.

**Table 1:** Demographic and clinical characteristics of the patients.

**Characteristic**	**Patients who present with obstructive jaundice following gastrectomy (*N =* 36)**
Age (years, mean ± SD)	63.3 (±11.0)
**Gender**	
Male, *n* (%)	30 (83.3%)
Female, *n* (%)	6 (16.7%)
BMI (kg/m^2^, mean ± SD)	19.5 (±3.1)
Body temperature (°C, median/range)	36.7 (36.0, 38.8)
**Bilirubin level (umol/L)**	
Total bilirubin level (umol/L, median/range)	127.1 (37.4, 488.9)
Direct bilirubin level (umol/L, median/range)	94.3 (20.1, 426.4)
**Tumor markers**	
Elevated CEA (*n*%)	16/31 (51.6%)
CEA level (ng/ml, median/range)	5.4 (0.9-231.1)
Elevated CA19-9 (*n*%)	21/28 (75.0%)
CA19-9 level (U/ml, median/range)	72.1 (3.1–1,666)
Elevated CA242 (*n*%)	8/20 (40.0%)
CA 242 level (U/m, median/range l)	8.0 (0.3–150)
Elevated CA125 (*n*%)	7/17 (41.2%)
CA125 level (U/ml, median/range)	16.6 (7.4–212.0)
**Inflammatory markers**	
hs-CRP (mg/l, median/range)	15.0 (0.8, 125.0)
PCT (ng/ml, median/range)	0.2 (0.07, 36.9)
**Imaging examination (** ***n*** **%)**	
Contrast-enhanced CT	30 (83.3%)
MRI/MRCP	18 (50.0%)
PET/CT	14 (38.9%)
**Type of gastrectomy (** ***n*** **%)**	
Subtotal gastrectomy with Billroth I	6 (16.7%)
Subtotal gastrectomy with Billroth II	15 (41.7%)
Subtotal/total gastrectomy with Roux-en-Y	9 (25.0%)
Subtotal gastrectomy (procedure unspecified)	6 (16.7%)
**Reason for subtotal gastrectomy (** ***n*** **%)**	
Gastric ulcer/perforation	10 (27.8%)
Gastric cancer	26 (72.2%)
Time from gastrectomy to obstructive jaundice (years, median/range)	3 (0.2–53.0)

Unless stated otherwise, data are numbers of patients, BMI, Body Mass Index; hs-CRP, High-sensitivity C-reactive protein; PCT, procalcitonin; CEA, carcinoembryonic antigen; CA 19-9, carbohydrate antigen 19-9; CA 242, carbohydrate antigen 242; CA125, carbohydrate antigen 125.

### 3.2 Etiology of obstructive jaundice after subtotal gastrectomy

Among the 36 patients with a history of subtotal gastrectomy, 27 (75.0%) had developed obstructive jaundice attributable to malignant lesions. Gastric cancer recurrence and/or metastasis was the leading cause (16 patients, 44.4%), followed by other types of malignancies within or adjacent to the intra-hepatic and extra-hepatic bile duct system (11 patients, 30.5%), which included 7 cases of pancreatic cancer, 2 cases of cholangiocarcinoma, 1 case of gallbladder cancer, and 1 case of ampullary carcinoma. Benign causes of obstructive jaundice comprised of 4 cases of inflammatory bile duct stricture (11.1%), 3 cases of choledocholithiasis (8.3%), and 2 cases of IgG4-related disease, both of which involved the intra-pancreatic segment of common bile duct (5.5%).

### 3.3 Imaging findings of contrast-enhanced CT, MRI/MRCP and PET/CT

In 30 patients with contrast-enhanced CT, lesions involving the intra-pancreatic segment of the common bile duct were observed in 15 cases (50.0%), while 8 (26.7%) cases had lesions involving the extra-pancreatic segment of the common bile duct. Contrast-enhanced CT detected masses in 19 (63.3%) patients, including 12 (40.0%) intra-pancreatic masses, 3 (10.0%) intra-hepatic masses, 3 (10.0%) extra-pancreatic/extra-hepatic bile duct masses, 3 (10.0%) hilar masses, and 3 (10.0%) masses at surgical anastomoses. Multiple abdominal masses were found in 5 (16.7%) cases. Three cases of biliary strictures (10.0%) and 2 cases of bile duct wall thickening (6.7%) was observed. A total of 11 (36.7%) patients had lymphadenopathy, including 7 (23.3%) with abdominal lymph node enlargement and 4 (13.3%) with retroperitoneal lymph node enlargement; 3 (10.0%) cases exhibited both abdominal and retroperitoneal lymph node enlargement. Additionally, 4 patients had ascites and 1 had peritoneal implants detected on CT. Two cases of bile duct stones (6.7%) and 1 (3.3%) case of pancreatic diffuse enlargement were observed, solely in patients with benign obstructive jaundice.

In 18 patients with MRI/MRCP, lesions involving the intra-pancreatic segment of the common bile duct were observed in 12 (66.7%) cases, while 4 (22.2%) cases with lesions involving the extra-pancreatic segment of the common bile duct. MRI/MRCP detected masses in 12 (66.7%) patients, including 6 (33.3%) intra-pancreatic masses, 2 (11.1%) intra-hepatic masses, 2 (11.1%) extra-pancreatic/extra-hepatic bile duct masses, 3 (16.7%) hilar masses, and 2 (11.1%) masses at surgical anastomoses. Multiple abdominal masses were found in 3 (16.7%) cases. Two cases of biliary strictures (11.1%) and 2 (11.1%) cases of bile duct wall thickening were observed. A total of 7 (50.0%) patients had lymphadenopathy, including 5 (35.7%) and 2 (11.1%) cases with abdominal lymph node enlargement and retroperitoneal lymph node enlargement, respectively; 1 (5.5%) case exhibited both abdominal and retroperitoneal lymph node enlargement. Additionally, 1 (5.5%) patient had ascites and 1 (5.5%) had peritoneal implants detected on MRI. Two cases of bile duct stones (11.1%) and 1 (5.5%) case of pancreatic diffuse enlargement were ob-served exclusively in patients with benign obstructive jaundice.

In 14 patients with PET/CT, lesions involving the intra-pancreatic segment of the common bile duct were observed in 9 (64.3%) cases, while 3 (21.4%) cases had lesions involving the extra-pancreatic segment. PET/CT detected abnormal hypermetabolic masses in 9 (64.3%) patients, including 8 (57.1%) intrapancreatic masses, 1 intrahepatic mass, 1(7.1%) extra-pancreatic/extra-hepatic bile duct mass, 1 (7.1%) hilar mass, and 1 (7.1%) mass at surgical anastomosis. In addition, hypermetabolic lymphadenopathy was found in 5 (35.7%) patients, including 4 (28.6%) and 1 (7.1%) cases of celiac and retro-peritoneal lymph nodes, respectively; 4 (28.6%) cases had both abnormal hypermetabolic abdominal and retroperitoneal lymph nodes.

### 3.4 Accuracy of lesion localization, characterization, and extent evaluation with different imaging modalities

The accuracy of lesion localization, characterization, and extent evaluation with CT, MRI, and PET/CT is shown in Table 2. Concerning localization of the obstruction site, contrast-enhanced CT achieved a sensitivity of 100.0%, with a specificity of 66.7%. MRI/MRCP achieved a sensitivity of 85.71% and a specificity of 100.0%. PET/CT achieved a sensitivity of 100.0%, with a specificity of 60.0%. The three imaging modalities didn't show significant difference in diagnostic accuracy of lesion location (*P* = 0.923).

**Table 2:** Lesion localization, characterization and extensiveness evaluation with contrast-enhanced CT, MRI, and PET/CT.

	**Contrast-enhanced CT**	**MRI/MRCP**	**PET/CT**	***P*-value**
**Localization of obstruction site (intra-pancreatic common**				
**bile duct/above)**				
Sensitivity	100.0%	85.71%	100%	0.176
Specificity	66.7%	100.0%	60.0%	0.358
Accuracy	85.2%	88.9%	84.6%	0.923
**Characterization of obstructive lesion (benign/malignant)**				
Sensitivity	56.5%	70.0%	80.0%	0.488
Specificity	42.8%	87.5.0%	75.0%	0.205
Accuracy	53.3%	77.8%	78.6%	0.132
**Evaluation of lesion extensiveness (local/metastatic)**				
Sensitivity	37.5%	60.0%	83.3%	0.178
Specificity	85.7%	92.3%	100.0%	0.778
Accuracy	60.0%^a^	83.3%^a, b^	92.8%^b^	0.036

The different letter represents a significant difference between the two groups (*p* < 0.05).

Concerning characterization of the lesion causing obstructive jaundice, contrast-enhanced CT had a relatively low sensitivity and specificity, of 56.5% and 42.8%, respectively. MRI/MRCP achieved a higher sensitivity of 70.0% and a specificity of 87.5%. PET/CT achieved an even higher sensitivity of 80.0%, with a specificity of 75.0%. Likewise, the three imaging modalities didn't show significant difference in diagnostic accuracy of lesion location (*P* = 0.132).

When it comes to the evaluation of disease extensiveness, contrast-enhanced CT had a sensitivity and specificity of 37.5% and 85.7%, respectively. MRI/MRCP achieved a higher sensitivity and specificity of 60.0% and 92.3%. PET/CT achieved high sensitivity and specificity of 83.3% and 100%, respectively. The accuracy of PET/CT in determining extensive disease was significantly higher compared to contrast-enhanced CT and MRI/MRCP (*P* = 0.036).

**Figure 2:**
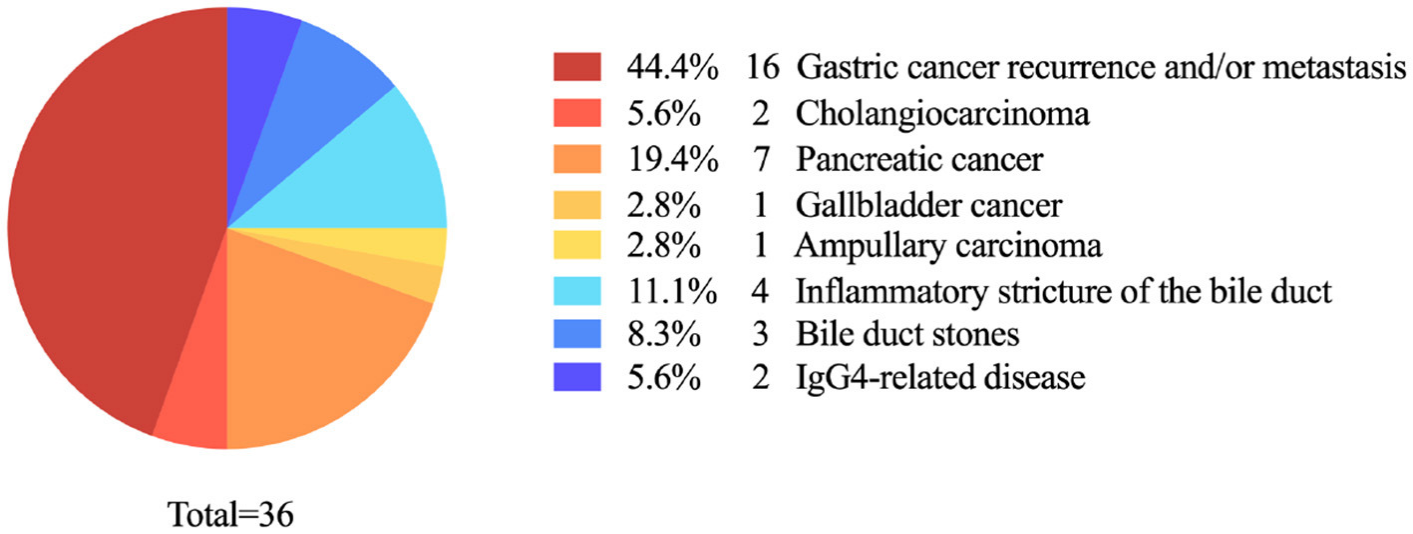
Etiology of obstructive jaundice after subtotal gastrectomy.

**Figure 3:**
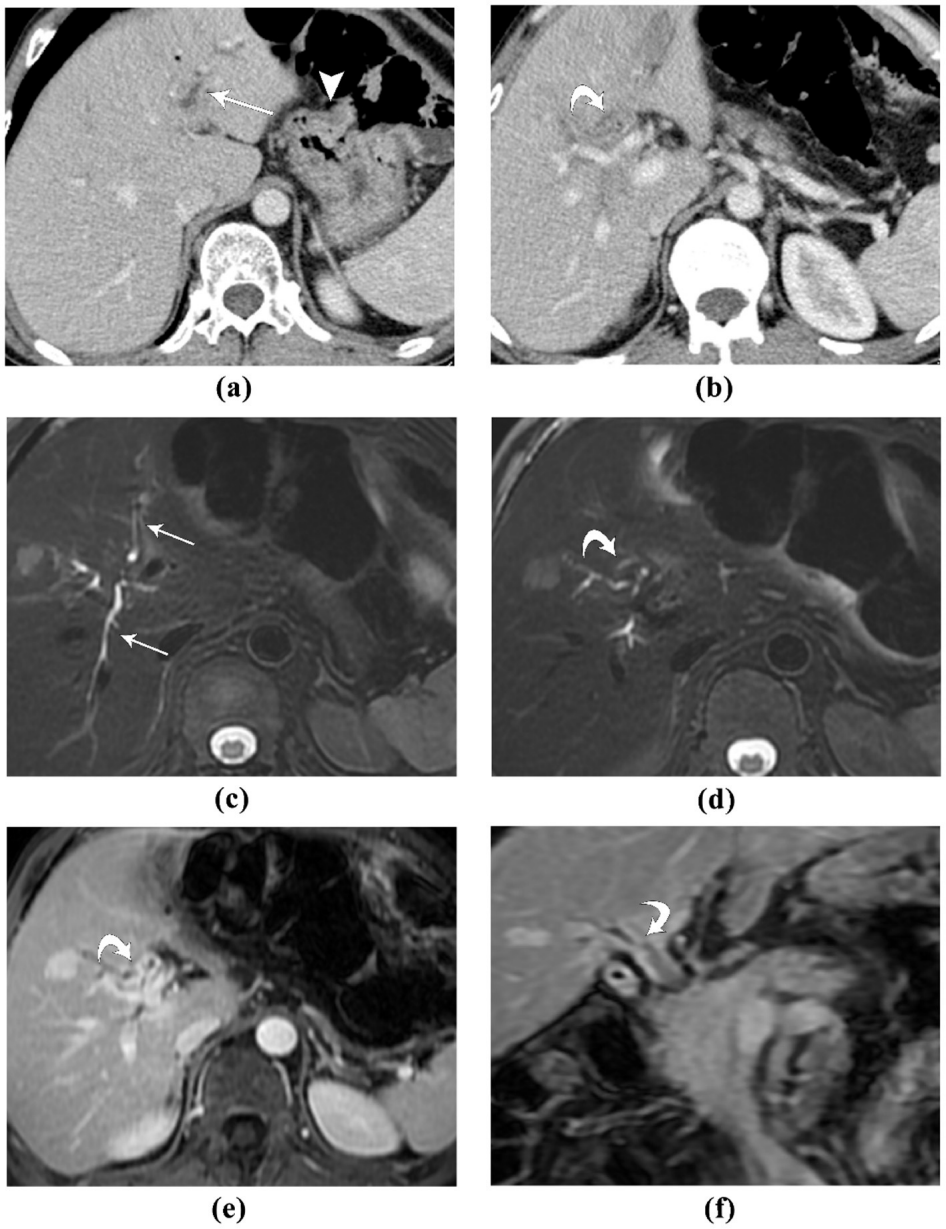
A 53-year-old male patient, who developed obstructive jaundice after 23 years of Bilroth II gastrectomy for peptic ulcer: **(A, B)** contrast-enhanced CT, portal venous phase images. The intra-hepatic bile ducts are dilated (*straight arrow*). The gastrojejunal anastomosis is seen (*arrowhead*). At the level of the bifurcation of left and right hepatic ducts, the bile duct wall is markedly thickened, with slight hyper-enhancement (*curved arrow*); **(C, D)** MR T2-weighted fat-saturated images. The intra-hepatic bile ducts are dilated (*straight arrow*), and the bile duct wall is thickened, which demonstrates intermediate to low T2 signal intensity; **(E, F)** contrast-enhanced MR, axial a coronal portal venous phase image. The hilar bile duct and the bifurcation of left and right hepatic duct demonstrated strong and homogeneous enhancement. This patient was diagnosed with IgG4-realted sclerosing cholangitis, which resolved after steroid therapy. Both contrast-enhanced CT and MRI depicts the location and extent of the lesion well, and correctly characterized it as benign.

**Figure 4:**
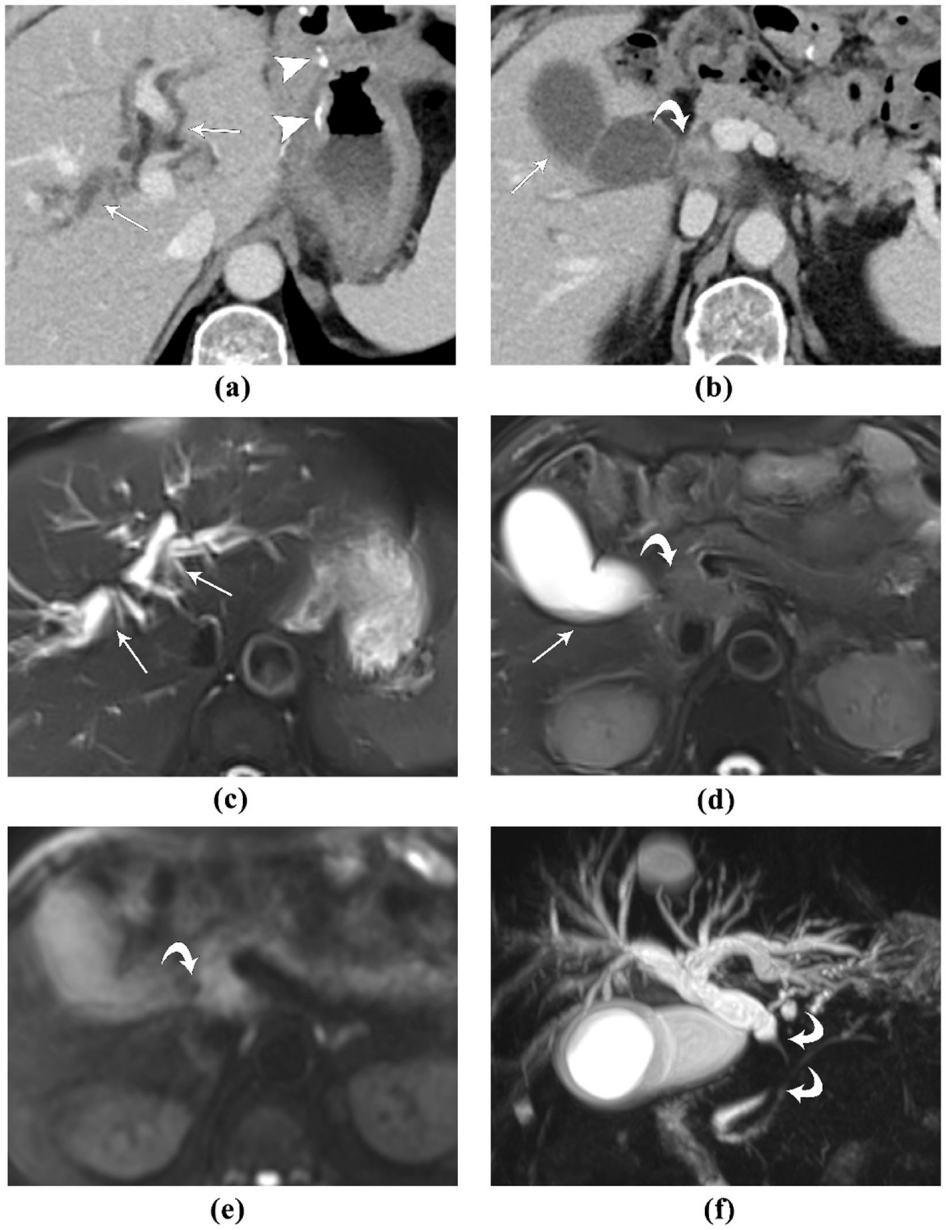
A 72-year-old male patient, who developed obstructive jaundice after 2 years of Bilroth II gastrectomy for gastric cancer: **(A, B)** contrast-enhanced CT, portal venous phase images. The high-density surgical sutures are seen (*arrowhead*). The lesion causing obstructive jaundice was initially missed. In retrospect, a soft-tissue mass with blurred margins and ring-like enhancement (*curved arrow*) is seen, which obstructs the extra-pancreatic extra-hepatic bile duct, causing the upstream biliary dilation (*straight arrows*); **(C, D)** MR T2-weighted fat-saturated images. The intra-hepatic bile ducts are dilated, and the gallbladder is distended with fluid-fluid level (*straight arrows*). The lesion causing obstructive jaundice was slightly hyper-intense to liver and pancreatic parenchyma (*curved arrow*); **(E)** MR diffusion-weighted image, the lesion was hyper-intense, suggestive of diffusion restriction. **(F)** MR cholangiopancreatography clearly depicts the occlusion of the extra-pancreatic extra-hepatic bile duct, and the upstream biliary dilation. This patient was diagnosed with gastric cancer metastasis to the bile duct. MRI depicts the location and extent of the lesion more clearly than contrast-enhanced CT.

In our study, the etiology of obstructive jaundice in some patients is IgG4-related disease. Both contrast-enhanced CT and MRI depicts the location and extent of the lesion well, and correctly characterized it as benign (Figure 2). For obstructive patients undergoing subtotal gastrectomy due to gastric cancer, the accuracy of different imaging modalities may vary (Figures 3, 4).

### 3.5 The efficacy of biliary drainage methods and post-operational complications

The majority of patients (25/36, 69.4%), underwent PTCD to relieve biliary obstruction followed by endoscopic retrograde cholangiopancreatography (ERCP) (9/36, 25.0%) (Table 3). Four patients underwent surgical intervention, including one case of open choledocholithotomy, one case of pancreaticoduodenectomy, one case of open tumor biopsy, and one case of choledocholithotomy followed by Roux-en-Y cholangiojejunostomy. Indications for surgery include bile duct stricture combined with bile duct stones after abdominal surgery, resectable mass lesions, unable to determine lesion characterization through imaging, and strong patient willingness to undergo surgery.

**Table 3:** Efficacy and complication rates of different biliary drainage methods.

**Biliary drainage**	**ERCP**	**PTCD**	**surgery**	***P*-value**
Implementation rate	9/36 (25.0%)^a, b^	25/36 (69.4%)^a^	4/36 (11.1%)^b^	< 0.001
Technical success	7/9 (77.8%)	24/25 (96.0%)	4/4 (100.0%)	0.265
Clinical success	6/7 (85.7%)	18/24 (75.0%)	3/4 (75.0%)	0.848
Complication incidence	1/9 (11.1%)	3/25 (12.0%)	1/4 (25.0%)	0.773

The different letter represents a significant difference between the two groups (*p* < 0.05).

ERCP led to the highest clinical success rate in managing obstructive jaundice, with 6 out of 7 patients (85.7%) obtaining clinical relief. PTCD and surgery followed with a clinical success rate of 75% (18/24 for PTCD and 3/4 for surgery). Three patients had complications after PTCD, including 2 cases of biliary leakage and 1 case of acute pancreatitis. One patient had complication after ERCP, presented as post-ERCP acute pancreatitis. One patient experienced bile leakage after explorative surgery.

After the intervention, 27 out of 35 patients (77.1%) had a decrease in total serum bilirubin of more than 30%. Median TBil level was 36.8 umol/L (range, 7.8–384.3 umol/L) and median DBil level was 28.5 umol/L (range, 2.7–341.8 umol/L). In 8 out of 11 patients (72.7%), CA19-9 levels decreased by < 30% or even increased.

### 3.6 Patient outcome

Regarding the short-term prognosis, follow-up data for at least 3 months was available for 30 out of the 36 patients. Among these, five patients died while 63.33% (19/30) experienced recurrence of obstructive jaundice or tumor progression within the follow-up timeframe. The disease-free survival rate was 36.67% (11/30), with the median duration of disease-free survival being 3 months (range, 0–14 months).

## 4 Discussion

Gastrointestinal reconstruction surgery significantly alters the structure and configuration of the gastrointestinal tract. Billroth I gastrectomy typically does not impact ERCP performance. Difficulty or failure of ERCP after Billroth II gastrectomy is primarily due to the long and angulated afferent loop caused by adhesion when the endoscope attempts to enter the afferent loop via the site of the gastrojejunal anastomosis (9). The reverse axis of common bill duct (CBD) also increases the difficulty of intubation. The Roux-en-Y anastomosis poses a challenge for endoscopic access due to the extended biliopancreatic limb length, hindering access to the biliary and biliopancreatic limbs and the ampulla of Vater, thereby making postoperative ERCP particularly challenging (10). Patients with surgically altered anatomy (SAA) have a higher risk of developing biliopancreatic complications. Even with the use of advanced device-assisted enteroscopy for ERCP, the risk of postoperative complications ranges from 0% to 19.5%, including perforation, bleeding, cholangitis, and pancreatitis (11).

Presently, most patients undergoing major gastrectomy in tertiary hospitals present with a history of gastric cancer, with a diminishing proportion of benign lesions like gastric ulcers and perforations. Consequently, vigilant monitoring is essential for patients with a history of gastric cancer, due to the risk of malignant obstructive jaundice from recurrence. In our cohort of 36 patients, averaging 63.3 years of age and predominantly with a history of gastric cancer (72.2%), the primary etiology of obstructive jaundice was malignant recurrence and metastasis of gastric cancer (47.5%), followed by other types of malignancies (30%). The high incidence of malignant obstructive jaundice and the limitations of postoperative endoscopic procedures underscore the importance of differentiating the nature of jaundice obstructions and determining whether the malignancy is a cancer recurrence or a primary secondary cancer in patients following gastrectomy. In the management of such patients, imaging plays an increasingly important role in early and accurate diagnosis of recurrence/progression throughout the entire follow-up period.

Various imaging modalities have unique strengths and limitations. The rapid acquisition and reduced artifact susceptibility of contrast-enhanced CT make it a valuable tool (12). However, its accuracy for lesion localization can be adversely affected in patients with prior biliary stent placement due to artifact interference (13). Studies have demonstrated that multidetector computed tomography (MDCT) shows significantly enhanced diagnostic accuracy in patients without biliary drainage procedures compared to those who have undergone such interventions. This difference may stem from the decompression-induced thinning of dilated intrahepatic bile duct walls, while the normal bile duct walls thicken due to inflammation after biliary drainage. Furthermore, artifacts from drainage tubes or stents can obscure hepatic hilum structures, making it more difficult to evaluate main or bilateral portal vein involvement (13). In our study, a total of three patients had contrast-enhanced CT scans performed after PTCD procedures, with two exhibiting diagnostic inaccuracies. Thus, performing CT and MRI/MRCP before PTCD or biliary stent insertion is recommended for obstructive jaundice patients to capture clear images of the dilated bile ducts and stenosis locations without the image quality being compromised by artifacts which complicates the assessment of secondary bile duct root involvement. Additionally, the limited sensitivity of CT in identifying lymph node and distant metastases can lead to erroneous preoperative assessments by missing small liver and peritoneal metastases (13). Our study also showed that the accuracy of contrast-enhanced CT in the diagnosis of benign and malignant lesions of obstructive jaundice is relatively low, mainly due to insufficient detection of distant metastasis and lymph nodes.

MRI/MRCP has higher sensitivity to biliary stones and soft tissue contrast, and can provide multiple different weighted contrast images, but it is susceptible to factors such as old age, patient cooperation, ascites, respiration, and artifacts. The sensitivity and specificity of MRCP for biliary stones are 89%−100% and 83%−100%, respectively (14), while the sensitivity of CT for common bile duct stones ranges from 72% to 88% (15). The reported sensitivity and specificity of MRCP compared to ERCP for the detection of bile duct malignancy are 81 and 100 % compared to 93 and 94 %, respectively (16). For cases of obstructive jaundice where immediate endoscopic intervention is not required, MRCP can almost serve as an alternative to diagnostic ERCP (2). However, MRCP encounters several diagnostic limitations. The Maximum-Intensity-Projection (MIP) technique may fail to reveal small filling defects due to the partial volume effect. Respiratory motion artifacts during MIP reconstruction can occur if the patient does not maintain a proper breath hold (17). Incomplete imaging may create confusion regarding ductal anatomy or disease, leading to the misinterpretation of pseudostrictures, such as those caused by pulsatile vascular compression simulating bile duct obstructions (18). Susceptibility artifacts from metallic foreign bodies or gastric-duodenal gas can also pose challenges. In addition, the constrained spatial resolution and static imagery of MRCP limit its effectiveness in differentiating between benign and malignant lesions.

The advantage of PET/CT over cross-sectional imaging is that it can detect extra-abdominal metastases and intra-abdominal lymph node metastases of FDG avid tumors. PET/CT is superior in detecting extra-abdominal and intra-abdominal lymph node metastases of FDG-avid tumors, although it has limitations in identifying small liver metastases and peritoneal carcinomatosis (19). In our study, PET/CT showed its superiority in detecting lesions characterization and extensiveness, mainly due to its sensitivity to distant metastasis and lymph nodes. However, PET/CT has limitations in detecting small-volume lesions and misinterpretation of local benign inflammatory changes as suspicious, which can affect the accuracy of local lesion staging (20, 21). There are many studies comparing the diagnostic ability between conventional imaging and FDG-PET/CT in recurrent pancreatobiliary tumor and has reported variable results. In a retrospective study by Banu et al. of 70 cases of pancreaticobiliary neoplasms, FDG-PET/CT and conventional cross-sectional imaging were shown to have very similar overall accuracy for detecting recurrence/progression of pancreaticobiliary tumors (84.2% and 87.1%, respectively) (19). Another study involving 25 cases of suspected recurrent pancreatic cancer revealed that FDG-PET outperformed CT and MRI in identifying local recurrence, although it was less sensitive than CT/MRI for liver metastases (22); this disparity may stem from the misclassification of benign inflammatory changes as potential malignancy (21). In Kumar et al.'s study of 24 patients, FDG-PET/CT demonstrated greater sensitivity, specificity, and accuracy in detecting recurrent gallbladder carcinoma compared to other modalities (93.7% vs. 87.5%, 100% vs. 50%, 95.8% vs. 75%, respectively) (23). Lee et al. found that 18F-FDG PET/CT was not more sensitive or specific than CT alone in detecting recurrent biliary tract cancer, but the combination of CT and 18F-FDG PET/CT significantly increased the sensitivity for detecting recurrence (24). Thus, a combination of imaging modalities can be used to avoid misdiagnosis and missed diagnoses. In our study, we compared the diagnostic accuracy of contrast-enhanced CT, MRI/MRCP, and PET/CT in localization, characterization, and extensiveness of obstructive jaundice lesions. All three techniques showed their reliability in localization diagnosis and exhibited comparable sensitivity, specificity, and accuracy (100%, 66.7%, 78.9% vs. 85.7%, 100.0%, 100.0% vs. 100.0%, 60.0%, 80.0%, respectively), which is basically consistent with previous studies. In the evaluation of disease extensiveness, the diagnostic accuracy of PET/CT was higher than that of contrast-enhanced CT and MRI/MRCP (92.8% vs. 60.0% vs. 83.3%, respectively). Due to the high costs and specialized operational requirements associated with PET-CT, its widespread adoption in resource-limited regions is constrained. PET-CT is typically not used as an initial diagnostic tool but rather for further assessment following a preliminary diagnosis. It is particularly deployed when other imaging modalities like ultrasound, CT, and MRI fail to precisely identify the location and nature of lesions, or when there is a need to assess distant involvement.

Biliary drainage (BD) is indicated for unresectable malignant lesions or as a preoperative measure for biliary decompression before curative surgery. ERCP and PTCD represent the most prevalent biliary drainage procedures, both established as effective and safe (3). A meta-analysis comparing ERCP and PTCD in biliary drainage for malignant obstructive jaundice reported higher technical success rates and a lower incidence of postoperative pancreatitis in the PTCD group (25). There was no significant difference in clinical efficacy, postoperative cholangitis, or bleeding rates between the two groups (24). In benign biliary disease, Kweon et al. compared the recurrence rates of choledocholithiasis after ERCP and PTCD in patients who had undergone gastrectomy and found that the ERCP group had a higher recurrence of choledocholithiasis than the PTBD group (24.4% vs. 10.0%) (26). This may be due to the fact that PTCD was less affected by changes in the gastroduodenal anatomy in patients who had undergone gastrectomy. In our study, PTCD and ERCP demonstrated a comparable technical success rate (96.0% vs. 77.8%), clinical relief rate (75.0% vs. 85.7%), and postoperative complication rate (12.0% vs. 11.1%). Subtle differences were noted in the spectrum of complications ensuing from the two interventions: One patient developed acute pancreatitis after ERCP, and three patients had complications after PTCD, including two cases of biliary fistula and one case of acute pancreatitis. Both methods had high technical success and clinical relief rates with low postoperative complications, indicating their efficacy. Our study showed that more patients took PTCD instead of ERCP (69.4% vs. 25%), indicating that PTCD is often the preferred method for biliary drainage for patients who have undergone gastrectomy due to anatomical changes.

Innovative alternatives to therapeutic ERCP or PTCD have been actively researched and applied. Device-assisted enteroscopy (DAE), including double balloon, short double balloon, single balloon, and spiral enteroscopy, has achieved high success rates in patients with surgically altered anatomy undergoing ERCP (11). Double-balloon enteroscopy-assisted ERCP (DBE-ERCP) boasts an overall success rate of 86% in patients with surgically altered anatomy (27). A meta-analysis of 15 studies with 461 patients undergoing single-balloon enteroscopy-assisted ERCP (SBE-ERCP) reported an overall procedural success rate of 61.7% (28). Methods that combine endoscopic ultrasound (EUS) or laparoscopy with ERCP can bypass the long route to the ampulla and provide direct access to the afferent loop. Gkolfakis et al. found that endosonography-directed transgastric-(EDGE) and laparoscopic-assisted (LA)-ERCP had technical success rates of 97.9% and 99.1%, respectively, in patient with SAA (29). In addition, novel intraductal ultrasonography has been applied to accurately measure the structure of the main pancreatic duct during pancreaticoduodenectomy, which helps to stratify the risk of postoperative complications in patients undergoing pancreatectomy and may have potential value in predicting patient prognosis (30). We look forward to broader application of advanced endoscopic techniques and surgical methods in clinical practice, improving the diagnosis and treatment of such patients.

Although both ERCP and PTCD can effectively alleviate the symptoms of obstructive jaundice in patients, patients often experience short-term progression, such as recurrence of obstructive jaundice or other manifestations caused by malignant tumor progression. This study showed that 5 people died (13.9%) during the follow-up period, and the disease-free survival rate was 36.7%, with a median disease-free survival of only 3 months, suggesting that patients with a history of subtotal gastrectomy often had a poor prognosis after they developed obstructive jaundice. At the beginning of treatment, most of these patients had lost the opportunity for surgery due to extensive tumor metastasis. ERCP and PTCD are only palliative treatments. We compared changes in tumor marker levels before and after interventional therapy and found that although biliary drainage effectively reduced bilirubin levels, there was rarely a reversal in tumor marker levels. In benign pancreaticobiliary diseases such as obstructive jaundice, cholestasis, biliary infection and pancreatitis, CA19-9 measurements may not accurately reflect the true secretion levels of the tumor, resulting in a high rate of false positives. Research by Daniele et al. indicates that an unchanged or >90 U/mL serum CA19-9 level after resolution of obstructive jaundice strongly suggests that the jaundice is caused by a malignant lesion. In our study, 72.7% (8 of 11) of patients had an unchanged or >90 U/mL CA19-9 level, supporting the diagnosis of malignant obstructive jaundice.

In this study, the causes of recurrent obstructive jaundice after gastrectomy were mostly malignant tumors, but there were also a few benign diseases (22.5%) with diverse causes, including 4 cases of inflammatory stenosis of the bile duct, 3 cases of biliary calculi, and 2 cases of IgG4-related disease. Research has demonstrated an increased incidence of gallstone subsequent to subtotal gastrectomy (31). This elevation in risk may be associated with reduced postoperative gastric motility leading to bile stasis, the effects of vagotomy, non-physiological reconstruction, infections within the bile duct, and modifications in the activity and secretion of cholecystokinin (32). IgG4-related disease has characteristic imaging findings and responds well to hormone therapy. The frequent recurrence of obstructive jaundice caused by gallstones and cholangitic stenosis requires multiple hospitalizations for the management of biliary obstruction or sustained PTCD drainage, significantly impairing the patient's life quality. Precise recognition of different benign diseases plays a pivotal role in facilitating prompt treatment and enhancing the prognosis.

There were several limitations in our study. First, its retrospective design led to incomplete clinical data, making it challenging to carry out extensive, long-term, and systematic follow-ups, thereby limiting the observation of disease progression and patient outcomes. In the diagnosis of obstructive jaundice, there is a lack of pathologic evidence in some patients. Second, as a single-center retrospective study with a modest sample size, the findings' applicability to a broader population requires verification through studies with larger cohorts. To date, there is a scarcity of large-scale research on obstructive jaundice patients post-subtotal gastrectomy, rendering our study an important reference point for future clinical guidelines. Third, the study exhibits selection bias. The included hospitalized patients generally presented with more severe conditions, suggesting that the outcomes documented in this study may not fully represent the broader patient population's experiences following subtotal gastrectomy.

This study provides a comprehensive understanding of the etiology, diagnosis, treatment, and prognosis of patients with obstructive jaundice after subtotal gastrectomy, thereby establishing a foundation for the diagnosis and treatment of such patients. It is anticipated that more advanced techniques will be adopted on a wider scale in clinical settings.

## 5 Conclusion

Gastric cancer recurrence and newly developed pancreaticobiliary malignancies were the main causes of obstructive jaundice in patients who had subtotal gastrectomy. PET/CT was superior to CT and MRI/MRCP in determining lesion extensiveness. PTCD was the preferred method for managing obstructive jaundice. Despite the effectiveness of interventions, a significant number of patients experienced short-term disease progression.

## Data Availability

The raw data supporting the conclusions of this article will be made available by the authors, without undue reservation.
